# Decreased expression of matrix metalloproteinase-1 in the maternal umbilical serum, trophoblasts and decidua leads to preeclampsia

**DOI:** 10.3892/etm.2015.2194

**Published:** 2015-01-20

**Authors:** CHUN-LEI DENG, SHENG-TAO LING, XUE-QIN LIU, YA-JUAN ZHAO, YUE-FENG LV

**Affiliations:** Department of Gynaecology and Obstetrics, Taihe Hospital, Hubei University of Medicine, Shiyan, Hubei 442000, P.R. China

**Keywords:** matrix metalloproteinase-1, tissue inhibitor of metalloproteinase-1, preeclampsia

## Abstract

The aim of the present study was to explore the levels of matrix metalloproteinase-1 (MMP-1) and tissue inhibitor of metalloproteinase-1 (TIMP-1) in the maternal umbilical serum, placenta and decidua of patients with preeclampsia compared with those in normotensive pregnant females. A total of 73 pregnant females were recruited as the test subjects, including 43 inpatients with hypertensive disorders in pregnancy and 30 normal pregnant females as the control. The 43 inpatients with hypertensive disorders in pregnancy included 18 patients with gestational hypertension, nine with mild preeclampsia and 16 with severe preeclampsia. MMP-1 and TIMP-1 ELISA kits were used to determine the MMP-1 and TIMP-1 levels in the umbilical serum of the parturient following delivery. MMP-1 and TIMP-1 expressed in the placenta and decidua of the parturient following delivery were evaluated using immunohistochemistry. MMP-1 and TIMP-1 were mainly located in cytotrophoblasts and syncytiotrophoblasts in the placenta and decidua. The levels of MMP-1 in the umbilical serum of the normal, gestational hypertension, mild preeclampsia and severe preeclampsia groups were 294.33±11.53, 247.78±20.32, 177.67±12.63 and 124.68±15.41 pg/ml, respectively, and there were significant differences between each two groups (P<0.05). The positive expression rate of MMP-1 in the placenta and decidua of patients with hypertensive disorders in pregnancy was lower than that of the controls (P<0.01 and P<0.01, respectively). However, no significant difference was identified between each two groups with regard to the levels of TIMP-1 in the umbilical cord and the positive rates in the placenta and decidua (P>0.05). Reduced MMP-1 levels in the umbilical serum, placenta and decidua were observed in women who developed preeclampsia.

## Introduction

Preeclampsia is a multi-system disorder of pregnancy characterized by hypertension, proteinuria and systemic vasoconstriction. The disorder is diagnosed in the latter half of pregnancy, affects ~5% of pregnant females and accounts for considerable mortality and morbidity (1). However its cause and pathogenesis are unclear. In previous years, vascular remodeling disorders of the uterine and placenta and placenta hypoperfusion have been generally recognized (2). It appears that when trophoblasts invade into spiral arteries insufficiently in the early stages of pregnancy, this impacts the process of vascular remodeling, resulting in ischemia and hypoxia of the placenta and causing hypertensive disease in pregnancy. The role of matrix metalloproteinases (MMPs) in the pathogenesis of hypertensive disorders in pregnancy has been the focus of much attention (3–6). Further studies have detected that MMP-1 is secreted from trophoblasts, which is an important factor in the regulation of trophoblast invasion (4,7–9).

MMPs are a type of proteinase, which can degrade the extracellular matrix (ECM) and remodel normal structure. Tissue inhibitors of metalloproteinases (TIMPs) are specific endogenous inhibitors that bind MMPs in a 1:1 stoichiometry, and their expression is regulated during development and tissue remodeling. TIMPs (21–29 kDa) have an N-terminal domain (125 amino acids) and a C-terminal domain (65 amino acids). The N-terminal domain folds as a separate unit and is capable of inhibiting MMPs (10). MMP-1 is an important member of the MMP family. The zymolytes of MMP-1 are collagen and metagelatin, which play major roles in trophoblast invasion (7). TIMP-1 is a natural inhibitor of MMP-1 (11).

However, there are few studies regarding MMP-1 and hypertensive disorders in pregnancy (1). This study explored whether reduced MMP-1 expression is associated with shallow trophoblast invasion and the pathogenesis of preeclampsia. MMP-1 and TIMP-1 protein expression in maternal umbilical serum, placenta and decidua from cases and controls were compared by ELISA and immunohistochemical analysis.

## Materials and methods

### Patient selection

Following approval by the ethics committee of Taihe Hospital (Shiyan, China) and informed consent from each patient, 73 pregnant females were recruited as the test subjects, including 43 inpatients with hypertensive disorders in pregnancy and 30 normal pregnant females as the control. The 43 inpatients with hypertensive disorders in pregnancy included 18 patients with gestational hypertension, nine with mild preeclampsia and 16 with severe preeclampsia. They all delivered in the obstetrical department of the Taihe Hospital between July 2011 and August 2012. All cases were single pregnancies where the patients were healthy and did not exhibit complications including hypertension, diabetes and heart, kidney or liver diseases prior to the study.

Gestational hypertension was defined by hypertension with systolic blood pressure ≥140 mmHg and/or diastolic blood pressure ≥90 mmHg, appearing for the first time after mid-pregnancy, without proteinuria. Mild preeclampsia was defined as hypertension with a systolic blood pressure ≥140 mmHg and/or a diastolic blood pressure ≥90 mmHg in association with proteinuria [24 h urinary protein >300 mg per 24 h or persistent 30 mg/dl (1+ on dipstick testing) in random urine samples] with or without edema. Severe preeclampsia was defined as hypertension with a systolic blood pressure ≥160 mmHg and/or a diastolic blood pressure ≥110 mmHg in association with proteinuria [24 h urinary protein >2 g per 24 h or persistent 200 mg/dl (2+ on dipstick testing) in random urine samples] with or without edema.

### Experimental methods

Hemostatic umbilical vein (5 ml) was removed rapidly following delivery, solidified under room temperature, then centrifuged at 5,000 × g for 10 min at 4°C and the serum was preserved at −80°C. Subsequent to delivery of the placenta, the maternal side of the placenta (1×1×1 cm) and the decidua were promptly removed. These were then rinsed three times with physiological saline and fixed immediately with formalin for 24–48 h, imbedded in paraffin, then cut into 3-μm slices.

Total MMP-1 and TIMP-1 levels in umbilical serum were measured using Human MMP-1/TIMP-1 PicoKine™ ELISA kits (rabbit anti-human; Boster Biological Engineering, Wuhan, China), according to the manufacturer’s instructions. Optical density was measured at 450 nm. The levels of MMP-1 and TIMP-1 in the placenta and decidua were detected by immunohistochemistry (streptavidin-biotin complex). Polyclonal (rabbit anti-human) antibodies against MMP-1 and TIMP-1 (diluted 1:200, Boster Biological Engineering) were used to assess the cellular expression of the protein. Double immunostaining was performed in an automated slide stainer following deparaffination in xylene, rehydration and heat-induced antigen retrieval at 37°C for 10 min in Tris-buffered saline at pH 6 (Dako, Glostrup, Denmark). Bovine serum albumin (2%) was added for 10 min to inhibit non-specific binding. A primary antibody mixture was added and the slides were incubated overnight at 4°C. The slides were incubated with secondary immunoglobulin G antibodies [tetramethyl rhodamine isothiocyanate-conjugated (goat anti-rabbit) antibody and fluorescein isothiocyanate-conjugated (goat anti-rabbit) antibody] at 1:200 dilutions for 30 min in a dark chamber. All sections were counterstained with DAPI and examined using an electron microscope (BX51; Olympus Corporation, Tokyo, Japan) at ×400 magnification. Negative controls were performed by omission of the primary antibodies as well as their substitution by isotype-matched rabbit serum.

The proportions of trophoblasts and deciduas expressing the MMP-1 protein were independently assessed by two pathologists, blinded to group status, subsequent to reaching an agreement on the inclusion criteria for positively stained cells. The positive or negative results were judged through the dye area, and the dye strength in the observed area. The dye area was evaluated on a scale of 0–3: 0, none; 1, ≤25%; 2, 26–49% and 3, ≥50%. The dye strength was evaluated on a scale of 0–2: 0, no dyeing; 1, moderate dyeing and 2, strong dyeing. The two points were added to assess the grades as follows 0 points is (−), 1–2 points is (+), 3–4 points is (++) and 5 points is (+++).

### Statistical analysis

All results were processed using SPSS software, version 17.0 (SPSS, Inc., Chicago, IL, USA). Statistical analyses of clinical data were performed using the unpaired two-sample Student’s t-test (two-sided) for continuous variables after testing for Gaussian distribution. The measurement data were examined by variance analysis or Fisher’s exact test. The enumeration data were examined by the χ^2^ test. A level of P<0.05 was considered to indicate statistical significance.

## Results

### Patient characteristics

The mean ages, gestational ages and infant birth weights of all subjects are listed in Table I. The difference in the age and gestational age of the subjects between the two groups revealed no statistical significance (t=1.589, P=0.116 and t=1.064, P=0.294, respectively). The differences in the birth weight among the two groups indicated significant differences (t=3.008, P=0.004).

### Serum MMP-1 and TIMP-1

The levels of MMP-1 and TIMP-1 in the serum of the umbilical cord are listed in Tables II and III, respectively. The levels of MMP-1 in the umbilical serum of the normal, gestational hypertension, mild preeclampsia and severe preeclampsia groups were 294.33±11.53, 247.78±20.32, 177.67±12.63 and 124.68±15.41 pg/ml, respectively, and there were significant differences between each two groups (P<0.05). However, the levels of TIMP-1 in the umbilical serum of the four groups were 1,304.20±69.66, 1,326.20±329.86, 1,340.11±547.05 and 1,363.00±71.50 pg/ml, respectively, and no significant difference was identified between each two groups regarding the level of TIMP-1 in the umbilical serum (P>0.05).

### MMP-1 and TIMP-1 expression in the placenta and decidua

**Figure 1:**
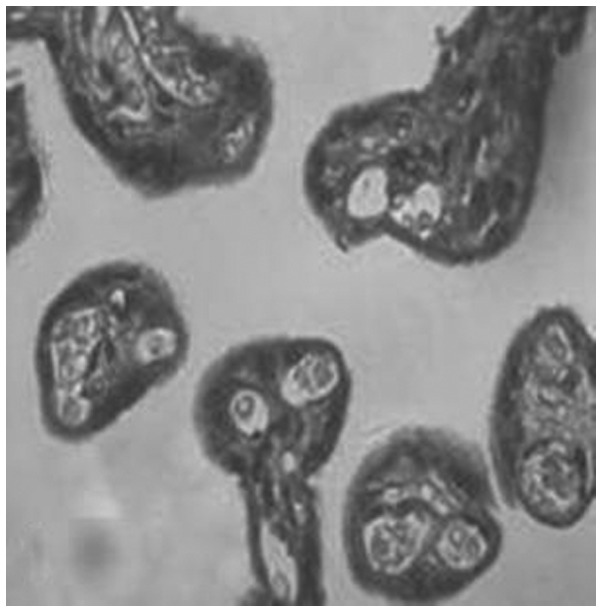
Immunohistochemical staining for matrix metalloproteinase-1 in a normal placenta. Magnification, ×400.

**Figure 2:**
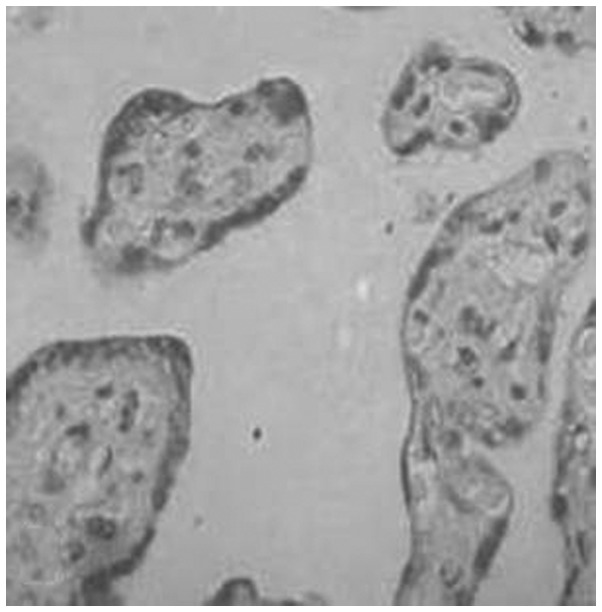
Immunohistochemical staining for matrix metalloproteinase-1 in the placenta of a patient with gestational preeclampsia. Magnification, ×400.

**Figure 3:**
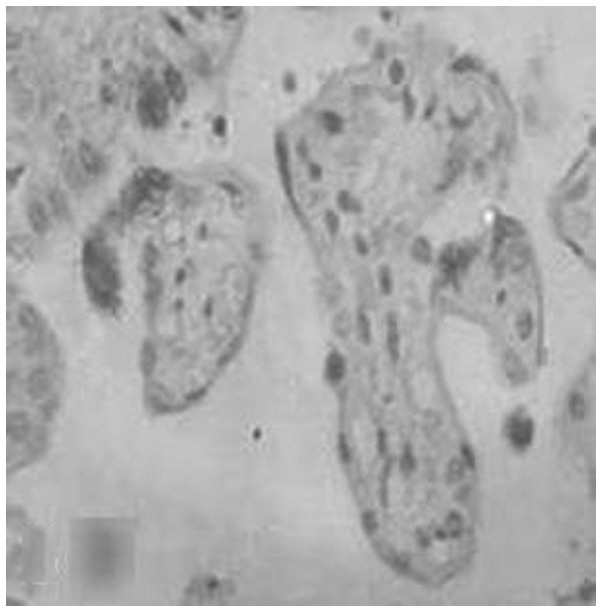
Immunohistochemical staining for matrix metalloproteinase-1 in the placenta of a patient with mild preeclampsia. Magnification, ×400.

**Figure 4:**
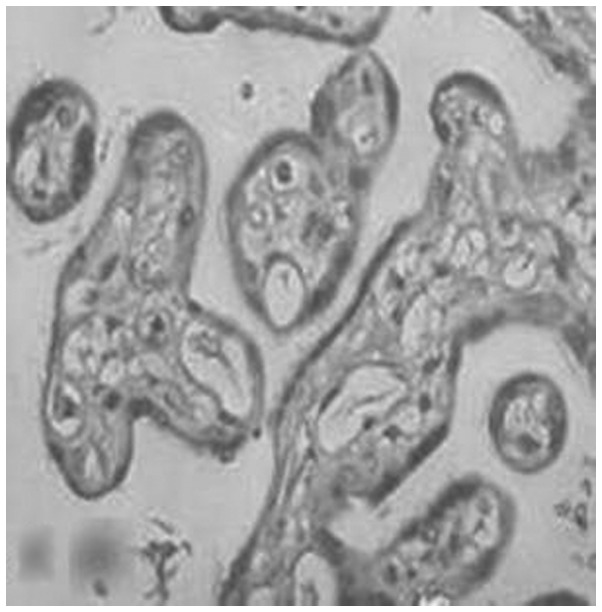
Immunohistochemical staining for matrix metalloproteinase-1 in the placenta of a patient with severe preeclampsia. Magnification, ×400.

**Figure 5:**
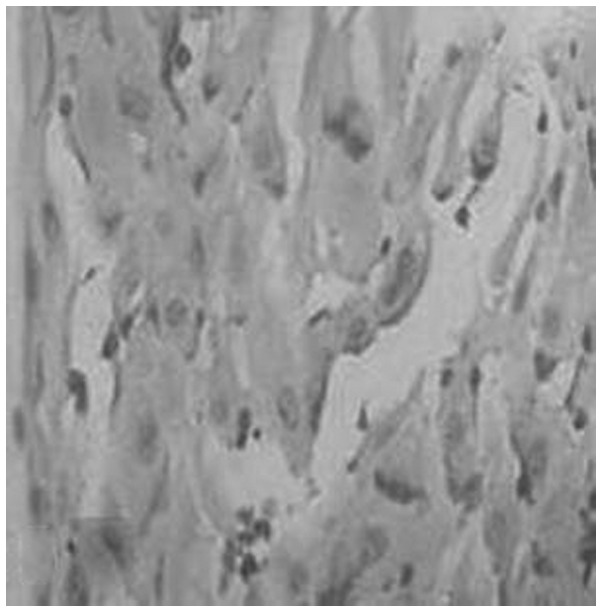
Immunohistochemical staining for matrix metelloproteinase-1 in a normal decidua. Magnification, ×400.

**Figure 6:**
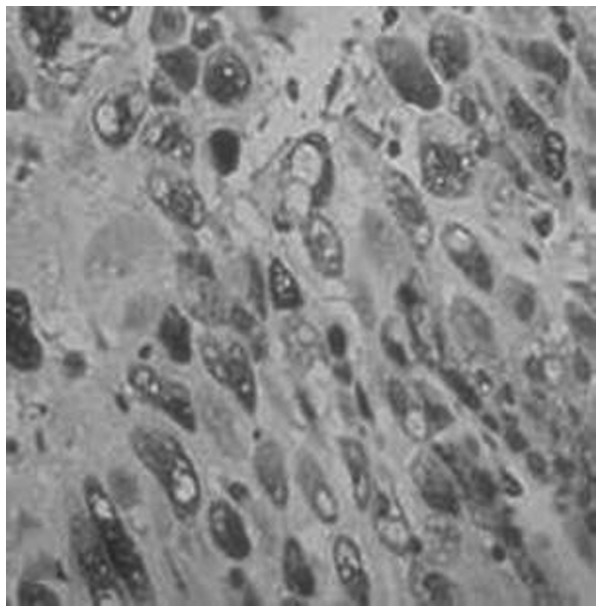
Immunohistochemical staining for matrix metalloproteinase-1 in the decidua of a patient with gestational preeclampsia. Magnification, ×400.

**Figure 7:**
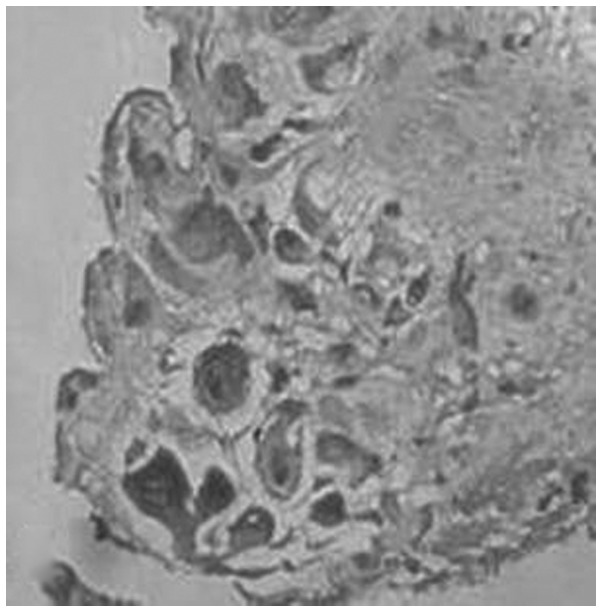
Immunohistochemical staining for matrix metalloproteinase-1 in the decidua of a patient with mild preeclampsia. Magnification, ×400.

**Figure 8:**
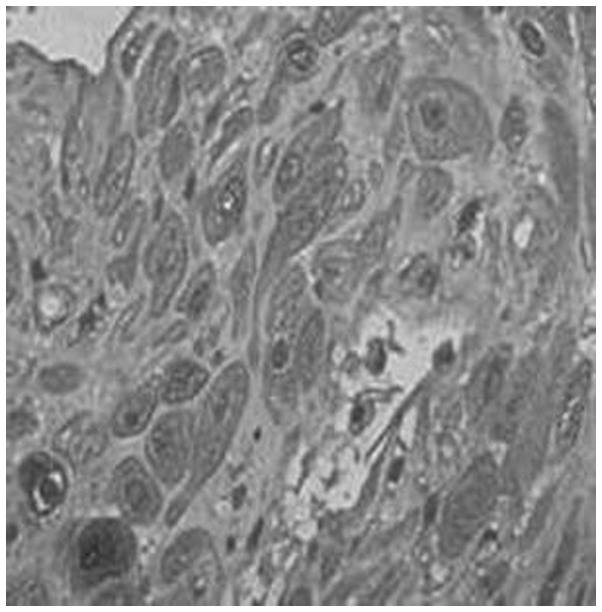
Immunohistochemical staining for matrix metalloproteinase-1 in the decidua of a patient with severe preeclampsia. Magnification, ×400.

The expression of MMP-1 and TIMP-1 was mainly located in the cytomembrane and the cytoplasm of the placental trophoblasts. They were weakly dyed in the endothelial cells of the capillaries and the stromal fibroblasts, moderately dyed in the cytomembrane and the cytoplasm of the decidua, and weakly dyed in the cytoplasm of the spiral artery. The positive rates of expression of MMP-1 in the placenta of the normal, gestational hypertension, mild preeclampsia and severe preeclampsia groups were 96.7, 77.8, 66.7 and 23.1%, respectively (Figs. 1–4, Table IV). There were significant differences in MMP-1 expression between each two groups (P<0.05). The positive rates of expression of MMP-1 in the decidua were 93.3, 77.8, 55.6 and 12.5%, respectively (Figs. 5–8, Table V). There were significant differences between each two groups (P<0.05).

**Figure 9:**
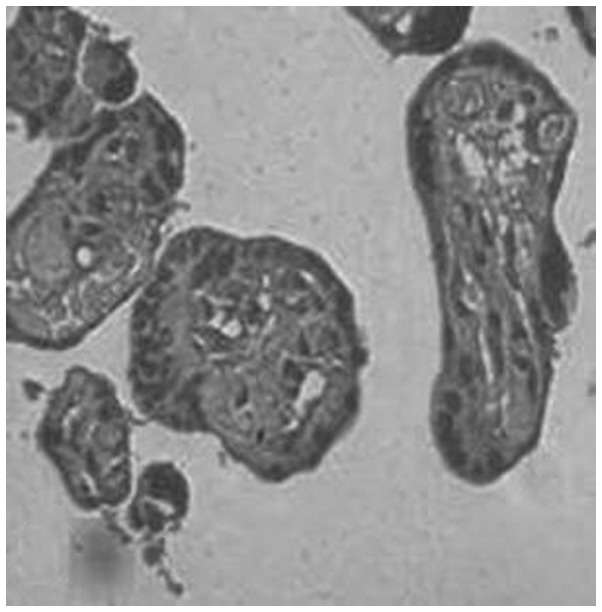
Immunohistochemical staining for tissue inhibitor of metalloproteinase-1 in the placenta of a patient with severe preeclampsia. Magnification, ×400.

**Figure 10:**
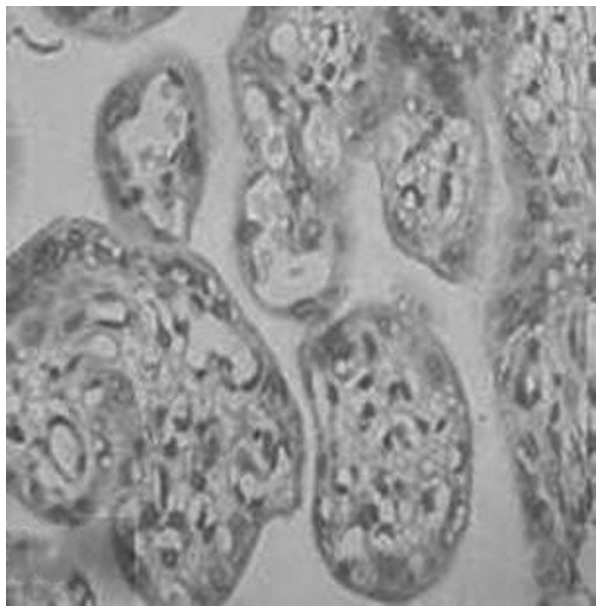
Immunohistochemical staining for tissue inhibitor of metalloproteinase-1 in a normal placenta. Magnification, ×400.

**Figure 11:**
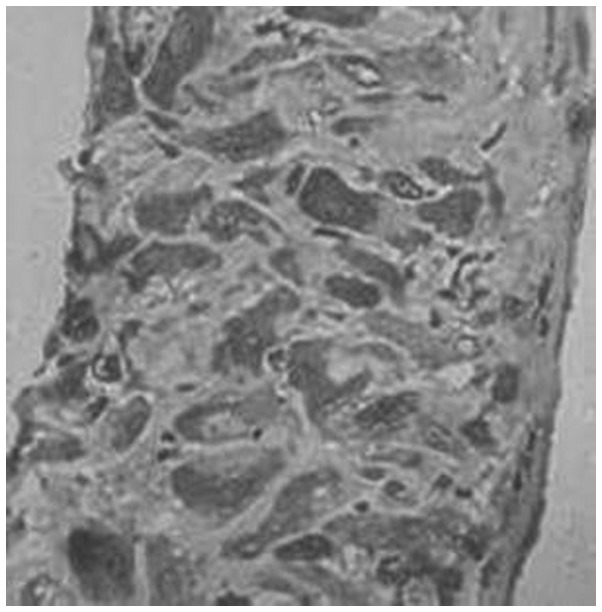
Immunohistochemical staining for tissue inhibitor of metalloproteinase-1 in the decidua of a patient with severe preeclampsia. Magnification, ×400.

**Figure 12:**
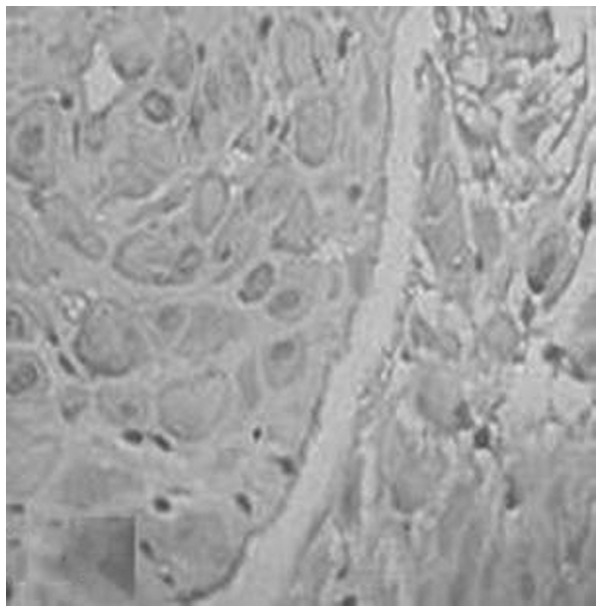
Immunohistochemical staining for tissue inhibitor of metalloproteinase-1 in a normal decidua. Magnification, ×400.

However, the positive rates of expression of TIMP-1 in the placenta of the normal, gestational hypertension, mild preeclampsia and severe preeclampsia groups were 56.7, 61.1, 66.7 and 75.0%, respectively (Figs. 9 and 10, Table VI). No significant differences were identified between each two groups (P>0.05). The positive rates of expression of TIMP-1 in the decidua of the four groups were 60.0, 61.1, 66.7 and 68.8%, respectively (Figs. 11 and 12, Table VII). No significant differences were identified between each two groups (P>0.05).

### Correlation

The levels of MMP-1 in the hypertensive disorders in the pregnancy and control groups exhibited positive correlations with the MMP-1 levels in the placenta (r=0.921, P<0.05), and also in the decidua (r=0.885, P<0.05). The levels of TIMP-1 in the hypertensive disorders in pregnancy and control groups exhibited positive correlations with the MMP-1 levels in the placenta (r=0.891, P<0.05) and the decidua (r=0.914, P<0.05).

## Discussion

In this study, investigation of protein expression at the maternal-fetal interface revealed that MMP-1 was decreased in the umbilical serum, placenta and decidua of the patients with preeclampsia compared with the controls. The proportions of MMP-1 to TIMP-1 in the umbilical serum, placenta and decidua were also all decreased. MMPs are a family of proteolytic enzymes that degrade various components of the ECM. MMP-1 is an important member, which particularly degrades interstitial collagen (12) and is abundant in tissues of the placenta and decidua. The invasive capacity of trophoblasts has been associated with their secretion of MMP-1 (13). TIMPs are specific endogenous inhibitors that bind MMPs in a 1:1 stoichiometry.

The present study revealed that MMP-1 and TIMP-1 were mainly expressed in the cytotrophoblasts and syncytiotrophoblasts of the placenta and decidua (Figs. 1–12). This was consistent with the aforementioned studies. In the process of embryo implantation and placentation, trophoblast invasion demands that they secrete hydrolysis enzymes effectively and degrade the major components of the ECM, including collagen, glycoproteins and proteoglycans. MMPs are effective hydrolyzing enzymes that are secreted by trophoblasts, and their expression is accurately regulated in time and space. In this process, trophoblasts invade the spiral arteries of the uterus and replace vascular muscle elastic membrane with fibrin, resulting in hemangiectasis, decreased vascular resistance and significantly increased blood flow. These physiological changes are termed vascular remodeling (14). From the present study, it appeared that MMP-1 secretion and subsequent ECM degradation occurred in the direction of invasion.

MMP-1 expression has also been shown to be crucial for the migratory capacity of mesenchymal stem cells (8). The present study has provided evidence suggesting that impaired trophoblast invasion in hypertensive disorders in pregnancy is associated with reduced MMP-1 levels in trophoblasts and decidual cells. There are at least three potential pathogenetic mechanisms by which MMP1 promotes trophoblast invasion (15). Firstly, MMP-1 could hydrolyze the basement membrane, interstitial decidua and vascular cavity surface, rendering these clear of physical barriers for trophoblast invasion. Secondly, MMP-1 may be associated with the apoptosis of decidual cells. Thirdly, MMP1 could also play a biological role through other proteins.

The results of the present study demonstrated that the expression levels of MMP-1 in the umbilical cord blood, placenta and decidua of patients with hypertension disorder in pregnancy were clearly lower than those in patients with normal pregnancy (P<0.05). With the aggravation of illness, MMP-1 expression reduced more markedly and the positive rates of MMP-1 and TIMP-1 dropped. It is hypothesized that in hypertensive disorders in pregnant patients, the trophoblasts were dysplastic, and the invasion ability was lower than that in patients at normal late pregnancy. The trophoblasts invaded the spiral arteries and uterine smooth muscle insufficiently (shallow placenta implantation). Therefore, the spiral arteries could not adapt to the physiological changes in pregnancy, which caused a reduction of the blood flow of the placenta, reduction of the oxygen content of the villi, villous ischemia and anoxia. The conclusion of the present study is consistent with the findings of Jurajda *et al* (16). All these factors may be associated with hypertension disorder in pregnancy. It is speculated that MMP-1 and TIMP-1 may be involved in the occurrence and development of hypertension disorders in pregnancy in every part of the maternal-fetal interface. However, this study was the first step in exploring the association between MMP-1 and preeclampsia. Further investigations at the RNA and DNA molecular level are required to provide more evidence regarding this association.

## Figures and Tables

**Table I:** Age, gestational age and birth weight in the two groups.

Group	Age (years)	Gestational age (weeks)	Birth weight (g)
Normal	27.77±4.09	37.27±2.27	3293.33±343.09
Experimental	29.51±4.94	36.78±1.25	2965.65±586.98
t	1.589	1.064	3.008
P-value	0.116	0.294	0.004

Data are presented as the mean ± standard error of the mean.

**Table II:** Levels of MMP-1 in serum of the umbilical cord.

Group	n	MMP-1 (pg/ml)
Normal	30	294.33±11.53
Gestational hypertension	18	247.78±20.32
Mild preeclampsia	9	177.67±12.63
Severe preeclampsia	16	124.68±15.41

Variance analysis indicates that there are statistical differences between every two groups (P<0.05). Data are presented as the mean ± standard error of the mean. MMP-1, matrix metalloproteinase-1.

**Table III:** Levels of TIMP1 in serum of the umbilical cord.

Group	n	TIMP-1 (pg/ml)
Normal	30	1304.20±69.66
Gestational hypertension	18	1326.20±329.86
Mild preeclampsia	9	1340.11±547.05
Severe preeclampsia	16	1363.00±71.50

Variance analysis indicates no statistical differences between every two groups (P>0.05). Data are presented as the mean ± standard error of the mean. TIMP-1, tissue inhibitor of metalloproteinase-1.

**Table IV:** Immunocytochemical distribution of matrix metalloproteinase-1 in the placenta.

Group	n	Matrix metalloproteinase-1				Positive rate (%)

		−	+	++	+++	
Normal	30	1	7	10	12	96.7
Gestational hypertension	18	4	6	5	3	77.8^a^
Mild preeclampsia	9	3	3	2	1	66.7^b^
Severe preeclampsia	16	13	2	1	0	23.1^c^

The χ^2^ test indicated that there were statistical differences between ^a^the gestational hypertension group and controls (χ^2^=4.301, P<0.05), ^b^the mild preeclampsia and gestational hypertension groups (P=0.009) and ^c^the severe preeclampsia and mild preeclampsia groups (P=0.025).

**Table V:** Immunocytochemical distribution of matrix metalloproteinase-1 in the decidua.

Group	n	Matrix metalloproteinase-1				Positive rate (%)

		−	+	++	+++	
Normal	30	2	6	9	9	93.3
Gestational hypertension	18	4	6	4	4	77.8^a^
Mild preeclampsia	9	4	2	2	1	55.6^b^
Severe preeclampsia	16	14	1	1	0	12.5^c^

The χ^2^ test indicated that there were statistical differences between ^a^the gestational hypertension group and controls (χ^2^=2.489, P<0.05), ^b^the mild preeclampsia and control groups (χ^2^=3.00, P<0.05) and ^c^the severe preeclampsia and mild preeclampsia groups (P=0.048).

**Table VI:** Immunocytochemical distribution of tissue inhibitor of metalloproteinase-1 in the placenta.

Group	n	Tissue inhibitor of metalloproteinase-1				Positive rate (%)

		−	+	++	+++	
Normal	30	13	8	6	3	56.7
Gestational hypertension	18	7	3	4	4	61.1^a^
Mild preeclampsia	9	3	2	2	2	66.7^b^
Severe preeclampsia	16	4	3	3	6	75.0^c^

The χ^2^ test indicated that there were no statistical differences between every two groups (P>0.05). Statistical differences were not identified between ^a^the gestational hypertension group and controls (*χ^2^*=4.301, P>0.05), ^b^the mild preeclampsia and gestational hypertension groups (P=0.778) and ^c^the severe preeclampsia and mild preeclampsia groups (P=0.877).

**Table VII:** Immunocytochemical distribution of tissue inhibitor of metalloproteinase-1 in the decidua.

Group	n	Tissue inhibitor of metalloproteinase-1				Positive rate (%)

		−	+	++	+++	
Normal	30	12	10	6	2	60.0
Gestational hypertension	18	7	4	4	3	61.1^a^
Mild preeclampsia	9	3	3	2	1	66.7^b^
Severe preeclampsia	16	5	4	3	3	68.8^c^

The χ^2^ test indicated that there were no statistical differences between every two groups (P>0.05). Statistical differences were not identified between ^a^the gestational hypertension group and controls (P=0.06), ^b^the mild preeclampsia and gestational hypertension groups (P=0.778) and ^c^the severe preeclampsia and mild preeclampsia groups (P=0.915).
